# Inhibition of Mogroside IIIE on isoproterenol-induced myocardial fibrosis through the TLR4/MyD88/NF-κB signaling pathway

**DOI:** 10.22038/IJBMS.2022.67908.14848

**Published:** 2023-01

**Authors:** Shi Yanan, Li Bohan, Sun Shuaifeng, Tian Wendan, Zizhe Ma, Liu Wei

**Affiliations:** 1 Department of Cardiology, the Fourth Affiliated Hospital, Harbin Medical University, Harbin, PR. China, 150001.; 2 Harbin Medical University, Harbin, PR. China, 150001.; 3 Heilongjiang Provincial Hospital, Harbin, PR. China, 150001.; 4 Department of Geriatric Cardiology, Guangdong Provincial People’s Hospital. Guangzhou, PR. China, 510080; # These authors contributed eqully to this work

**Keywords:** Cytokine, Inflammation, Myeloid differentiation- factor 88, Myocardial fibrosis, NF-κB, Toll-like receptor 4

## Abstract

**Objective(s):**

To investigate the effect of mogroside IIIE (MGIIIE) on isoproterenol (ISO)-induced myocardial fibrosis and explore its possible mechanisms.

**Materials and Methods:**

Forty C57BL/6 male mice (6-8 weeks) were randomly divided into a control group (n=10), model group (n=10), low MGIIIE dose group (n=10), and high MGIIIE dose group (n=10). Myocardial fibrosis was established by subcutaneous ISO injection. After 2 weeks of continuous gastric administration of MGIIIE, the cardiac structure was evaluated by echocardiography. Myocardial inflammation and fibrosis were evaluated by histology examination. Toll-like receptor 4 (TLR4), myeloid differentiation factor 88 (MyD88), p-IκBα, p-NF-κB, transforming growth factor β1 (TGF-β1), and α-smooth muscle actin (α-SMA) expression were detected by western blot. Inflammatory cytokines (IL-1β, IL-6, and TNF-α) in the serum were examined by ELISA. In the *in vitro* study, Ang II (1 μmol/l) was used to stimulate the fibroblasts, then inflammation and fibrosis index were detected.

**Results:**

MGIIIE inhibited inflammation and fibrosis and down-regulated TLR4, MyD88, TGF-β1, and α-SMA expression in the myocardium. In the *in vitro* study, MGIIIE ameliorates the deposition of Col Ш and Col I and decreases the release of inflammatory cytokines. MGIIIE increased p-IκBα and reduced p-NF-κB expression both *in vivo* and *in vitro*.

**Conclusion:**

MGIIIE plays a role in anti-myocardial fibrosis, by inhibiting TLR4/MyD88/NF-κB signaling expression, and decreasing inflammatory cytokine release. MGIIIE may represent a novel therapeutic strategy for treating cardiac fibrosis.

## Introduction

Myocardial fibrosis (MF) refers to the excessive aggregation of collagen fibers in the myocardial extracellular matrix and a significant increase in collagen concentration or composition (1). MF widely exists in a variety of cardiovascular diseases. It is a response of the myocardium to pressure overload, inflammatory reaction, and a variety of damage-related factors (2). Damage-related molecular pattern (DAMP) molecules include a class of substances that are released into the intercellular space or blood flow after the body is stimulated by injury, hypoxia, or inflammation. Previous studies have confirmed that DAMPs play a crucial role in promoting the occurrence and development of MF (3-5). 

Both the innate immune system and the adaptive immune system recognize exogenous DAMPs mainly through toll-like receptors (TLRs) (6-9). TLR is a pattern recognition receptor. DAMPs bind to and activate intracellular signal transduction cascades with the help of adaptor proteins, resulting in changes in gene expression and various cellular activities (10-12). Necrotic cells have been shown to activate TLR4 signaling in antigen-presenting cells (APCs) in the absence of any foreign microbial products (13). In previous studies, TLR4 activation has been shown to promote the progression of liver fibrosis (14). The TLR4/MyD88/NF-κB pathway leads to increased sensitivity of hepatic stellate cells (HSC) towards TGF-β1-induced signaling and allows unlimited activation and differentiation of fibroblasts into the extracellular matrix (ECM) producing myofibroblasts (15-17). 

*Siraitia grosvenorii* is a unique economic medicinal plant in China, which has the effects of clearing heat and diminishing inflammation (18-21). As the main active ingredient of siraitoside, siraitoside IIIE (MGIIIE) has been proven to down-regulate the production of inflammatory mediators related to the TLR4 signal transduction pathway. It is speculated that MGIIIE may inhibit tissue inflammation and fibrosis (22). Whether MGIIIE inhibits abnormal TLR4 activation and downstream inflammatory signaling to prevent MF remains unclear. In this study, we used an ISO-induced mouse myocardial injury model, a typical animal model of injury-related molecular patterns, to evaluate the effect and possible mechanism of MGIIIE-mediated protection against inflammation and myocardial fibrosis.

## Materials and Methods

***In vivo study***

*Animals and treatment*

The procedure for experimental animals was approved by the Animal Care Committee of Guangdong Provincial people’s hospital and complied with the guidelines for the care and use of experimental animals (KY-Q-2022-416). Male C57BL/6 mice (6-8 weeks old; weight 20 g±2 g) were purchased from Vital River Experimental Animal Technology Co., Ltd. (Beijing, China). The mice were randomly divided into four groups: 1) control group (n=10); 2) model group (n=10); 3) low dose MGIIIE group (n=10); and 4) high dose MGIIIE group (n=10). An MF model was induced by ISO. The mice were subcutaneously injected with the first dose of 40 mg/kg of ISO, 20 mg/kg on the second day, 10 mg/kg on the third day, and 5 mg/kg on the fourth day. This dose (5 mg/kg) was used subcutaneously for another 10 days (23). The control group received a subcutaneous injection of normal saline, whereas the low dose MGIIIE group (1 mg/kg) and the high dose MGIIIE group (10 mg/kg) received an intragastric administration of MGIIIE for 14 days.

*Assessment of cardiac structure and function by echocardiography*

On day 15, the mice were sedated intraperitoneally with 5% phenobarbital (0.1 ml/10 g), then fixed in the supine position. The chest was shaved with scissors. A PHILIPS-EPIQ 5 transthoracic echocardiogram (Bothell, WA, USA) was obtained by experienced ultrasound doctors using an S12-4 MHz imaging linear scan probe transducer. Left ventricular internal diameter at end-diastole (LVIDd) and LVEF were measured. The percentage of fractional shortening (FS) was then calculated. Peak mitral inflow E (mm/s) and A (mm/s) velocity waves on pulsed-wave Doppler were measured from the apical four-chamber view. The ratio of peak flow velocity across the mitral annulus during early and late diastole (E/A) was calculated from these values as a metric of diastolic function.

*Histopathological staining*

Apical tissue samples were dehydrated, embedded in paraffin, and cut into 5-μm-thick sections. These sections were stained with HE and Masson trichrome staining to assess histopathological changes and collagen deposition. The photographs were obtained with a light microscope. The number of inflammatory cells (blue, hematoxylin nuclei) was counted by quantifying 5 images/section using Image Lab software. The area percentage of collagen deposition was analyzed by the ratio of collagen deposition area to the total myocardial area.

*Western blot*

The cardiac tissue was washed twice with cold PBS and cleaved in a lysis buffer. From each sample, 20 μg of protein was separated by 12% SDS-polyacrylamide gel electrophoresis and transferred to a polyvinylidene fluoride membrane. Next, the membrane was incubated with primary antibodies against TLR4, MyD88, p-IκBα, p-NF-κB, TGF-β1, and α-SMA at 4 ^°^C overnight. Appropriate HRP-bound secondary antibodies were used to amplify the primary antibody signal. The gray levels of the target protein and β-actin bands were analyzed with Image Lab software and the relative expression of the target protein was calculated.

***In vitro study***

*Cell Culture and Treatments*

Primary cultures of neonatal mouse fibroblasts were prepared from SD mice by dissociating neonatal (1-3 days) hearts. Cardiac fibroblasts were expanded and passed twice before the assay to remove endothelial cell contamination. The cellular proliferation rate was evaluated through CCK-8 assay.

Ang II (1 μmol/l) was used to stimulate the fibroblasts. The optimal concentration of MGIIIE was screened in the pre-experiments, in which different concentrations of MGIIIE were used. The cells were incubated with an MGIIIE solution (0, 25, 50, 100, and 200 μmol/l) for 24 hr. It was confirmed that 100 μmol/l MGIIIE exhibited the most obvious effect on inhibiting the proliferation of myocardial fibroblasts. Then fibroblasts were incubated with 100 μmol/l MGIIIE and the control group did not receive any treatment. The cells in each group were incubated for 48 hr.

*Cell counting Kit-8 (CCK-8) Assay*

Cell proliferation was determined by CCK8 assay. 100 μl of counted cell suspension were inoculated into a 96-well plate. After the cells were completely attached to the wall, added 10 μl AngII and AngII plus MGIIIE mixture into the 96-well plate into 3 groups, and continued to incubate for 48 hr. Then added 1/10 CCK8 solution to each well and incubated for 4 hr. The optical density at 450 nm was detected by a microplate tester, and the cell proliferation rate was estimated by the optical density ratio between the treatment group and the blank control group.

*Western blot analysis*

Western blot analysis was performed as described previously (24, 25). Briefly, fibroblast proteins were separated by 10% SDS-PAGE and transferred to PVDF membranes (Millipore, USA). The membranes were blocked in 5% skim milk powder or BSA for 1 hr and incubated overnight at 4 ^°^C with the following primary antibodies: TGF-β1 (1:500), p-IκBα (1:500), p-NF-κB (1:500), Col I (1:1000), Col III (1:1000), and β-actin (1:1000). The membranes were subsequently incubated with the corresponding HRP-tagged secondary antibody for 1 hr at room temperature.

*RNA exaction and qRT-PCR*

qRT-PCR analysis was used to detect the levels of α-SMA, Col I, and Col III mRNA expression in fibroblasts. The total RNA was extracted from fibroblasts using Trizol reagent (Invitrogen Life Technologies, USA), reverse transcribed into complementary DNA (cDNA) using the TransScript First-strand cDNA Synthesis Kit (TOYOBO, Japan), and stored at -80 ^°^C until reverse transcription. The relative gene expression was quantified by q-PCR using SYBR® Premix Ex Taq™ (TaKaRa, China) in StepOne™ Real-Time PCR (Life Technologies, USA). In each reaction, 0.5 μg total RNA was reverse transcribed under the following PCR conditions: 94 ^°^C for 2 min, followed by 40 cycles at 94 ^°^C for 15 sec, 58 ^°^C for 30 sec, and 72 ^°^C for 30 sec, with a final extension at 72 ^°^C for 10 min. The primers for qRT-PCR were listed in Table 1. 

*Cytokine quantification using an enzyme-linked immunosorbent assay (ELISA)*

The concentration of IL-1β, IL-6, and TNF-α in the mouse serum and culture supernatants was determined using a commercial ELISA kit according to the manufacturer’s instructions (Elabscience, Wuhan, China). The optical density of the microplate was read at 450 nm.

***Statistical analysis***

GraphPad software was used for drawing and *SPSS20.0* software was used to statistically analyze the data. The measurement data were expressed in terms of the mean±standard deviation (deviation x±s). A single-factor analysis of variance (ANOVA) was used for comparisons between groups, and an LSD-*t* test was used for pairwise comparisons. *P*<0.05 indicated a statistically significant difference.

## Results

***Echocardiography***

**Figure 1:**
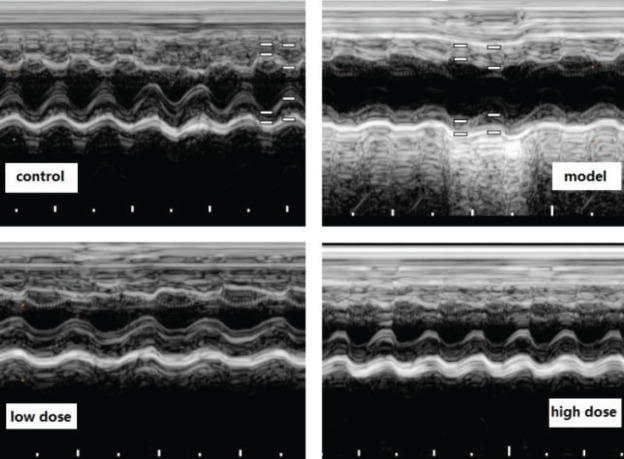
Typical pictures of echocardiography results for the *in vivo* study

After 2 weeks of MGIIIE administration, the cardiac structure and function were improved, cardiac cavity size was reduced, and systolic function and diastolic function were relieved (Figure 1, Table 2).

***HE and Masson’s trichrome staining***

**Figure 2:**
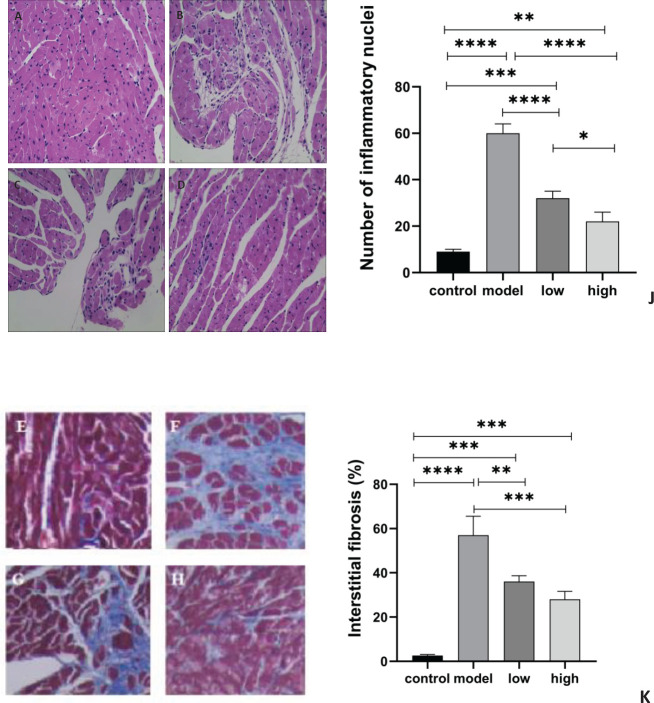
Histopathological examination results in each group for the *in vivo* study. A-D: HE staining results. (A) control group (n=10). (B) model group (n=6). (C) low dose MGIIIE group (n=5). (D) high dose MGIIIE group (n=5). (J) number of inflammatory nuclei. E-H: Masson staining results. (E) control group. (F) model group. (G) low dose MGIIIE group. (H) high dose MGIIIE group. (K) the percentage of myocardial area infiltrated by fibrosis

In the control group, the cardiomyocytes appeared uniform and tidy, without the presence of inflammatory cell infiltration or cardiomyocyte necrosis. The myocardial tissue in the model group exhibited a disordered structure, with infiltration of inflammatory cells and proliferation of fibrous tissue. Compared with the model group, there was moderate myocardial inflammation and fibrosis in both MGIIIE groups (Figure 2).

***Expression of TLR signal pathway proteins***

The results showed that TLR4, MyD88, and p-NF-κB-p65 expression was elevated in the model group. However, MGIIIE reduced the increased expression of TLR4, MyD88, and ISO-induced p-NF-κB-p65 expression. MGIIIE also increased the level of p-IκBα, which decreased following ISO treatment. Compared with the control group, the level of TGF-β1 and α-SMA protein expression in the model group was increased. MGIIIE inhibits TGF-β1 and α-SMA expression (Figures 3-1 and 3-2).

***qRT-PCR results***

A qRT-PCR analysis was performed on the cultured fibroblasts to determine the level of α-SMA, Col I, and Col III mRNA transcription. As shown in Figure 4, MGIIIE treatment could reduce the above-related levels of target mRNA expression (Figure 4).

***Expression of inflammation- and fibrosis-related proteins***

Figure 5 presents the expression of fibrosis-related proteins. The statistical results revealed that MGIIIE could reduce the levels of TGF-β1, Col I, and Col III protein expression, which were increased by Ang II stimulation *in vitro*. There were low levels of IκB phosphorylation in AngII-treated fibroblasts and MGIIIE treatment increased the level of p-IκB. MGIIIE treatment decreased the expression of NK-κB stimulated by Ang II.

***Effects of MGIIIE treatment on the expression of proinflammatory cytokines***

IL-1, IL-6, and TNF-*α* are important proinflammatory mediators, which are associated with the inflammatory response. To further determine whether MGIIIE inhibited ISO-induced inflammation *in vivo*, we used an ELISA to detect changes in the mouse serum. Compared with the control group, increased levels of IL-1β, TNF-*α,* and IL-6 were found in the ISO groups. However, these changes were ameliorated by MGIIIE treatment. In the* in vitro* study, we also found that MGIIIE pretreatment effectively inhibited Ang II-induced augment of IL-1β, TNF-*α,* and IL-6 in cultured fibroblasts (Figure 6). 

**Table 1:** Primers for qRT-PCR of Col I, Col III and α-SMA in the *in vitro* study

Groups	LVIDd (mm)	FS(%)	EF(%)	E/A ratio
Control (n=10)	46±2 ^a^^,^^b^	38±3 ^a^^,^^b^	72.1±5 ^a^^,^^b^	0.7±0.2 ^a^
Model (n=6)	65±3	28±4	52.7±6	1.2±0.3
Low-dose MGIIIE (n=5)	58±1 ^a^	35±4 ^a^	60.4±4 ^a^	0.8±0.3 ^a^
High-dose MGIIIE (n=5)	53±2 ^a^^,^^b^	38±5 ^a^^,^^b^	65.9±4 ^a^^,^^b^	0.8±0.1 ^a^

**Table 2:** Echocardiography results

Gene Primer sequence
collagen1-F GCTCCTCTTAGGGGCCACT collagen1-R ATTGGGGACCCTTAGGCCAT
collagen III-F CTGTAACATGGAAACTGGGGAAA collagen III-R CCATAGCTGAACTGAAAACCACC
α-SMA-F CCCAGACATCAGGGAGTAATGG α-SMA-R TCTATCGGATACTTCAGCGTCA
GAPDH-F AGGTCGGTGTGAACGGATTTG GAPDH-R GGGGTCGTTGATGGCAACA

Compared with the model group, ^a ^*P<*0.05. Compared with Low-dose MGIIIE group, ^b ^*P<*0.05

At the beginning of the experiment, there were 10 mice in each group. On day 14, the remaining numbers in each group were control group, n=10; model, n=6; low dose, n=5; high dose, n=5

LVIDd: Left ventricular internal diameter at end-diastole; FS: fractional shortening; EF: ejection fraction

**Figure 3-1:**
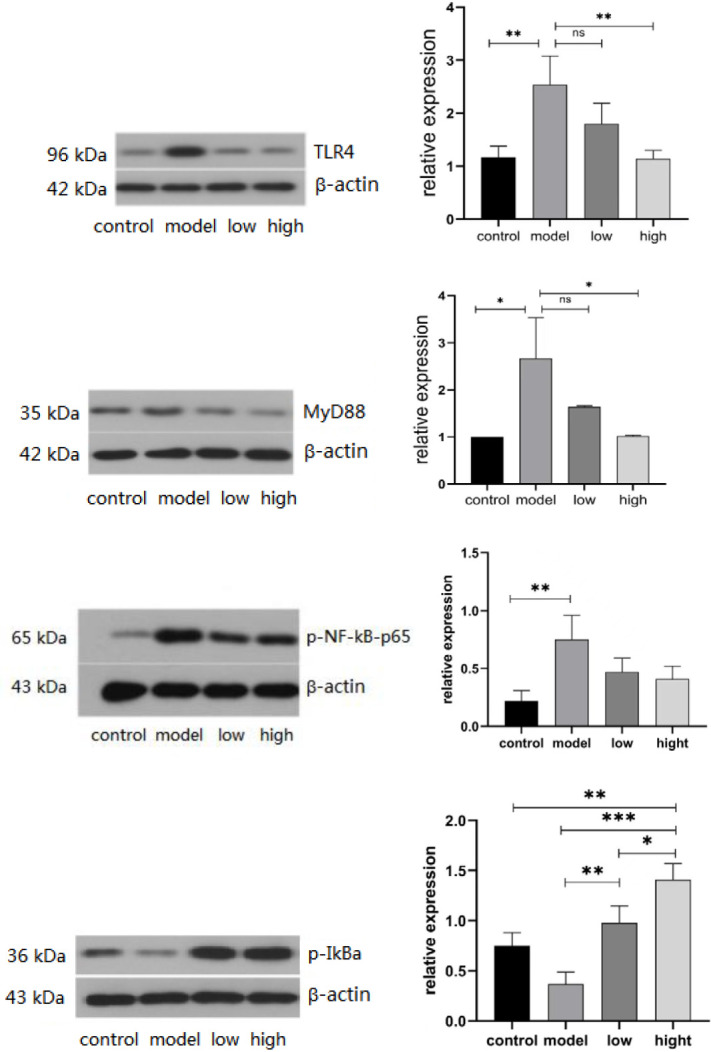
Western blot analysis results of TLR4/MyD88/NF-κB and IκB. Control group (n=10); model group (n=6); low dose MGIIIE group (n=5); high dose MGIIIE group (n=5)

**Figure 3-2:**
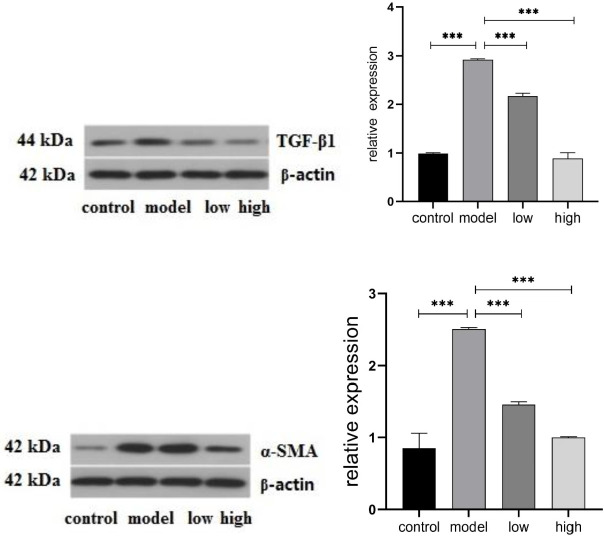
Western blot analysis of TGF-β1 and α-SMA protein results. Control group (n=10); model group (n=6); low dose MGIIIE group (n=5); high dose MGIIIE group (n=5)

**Figure 4:**
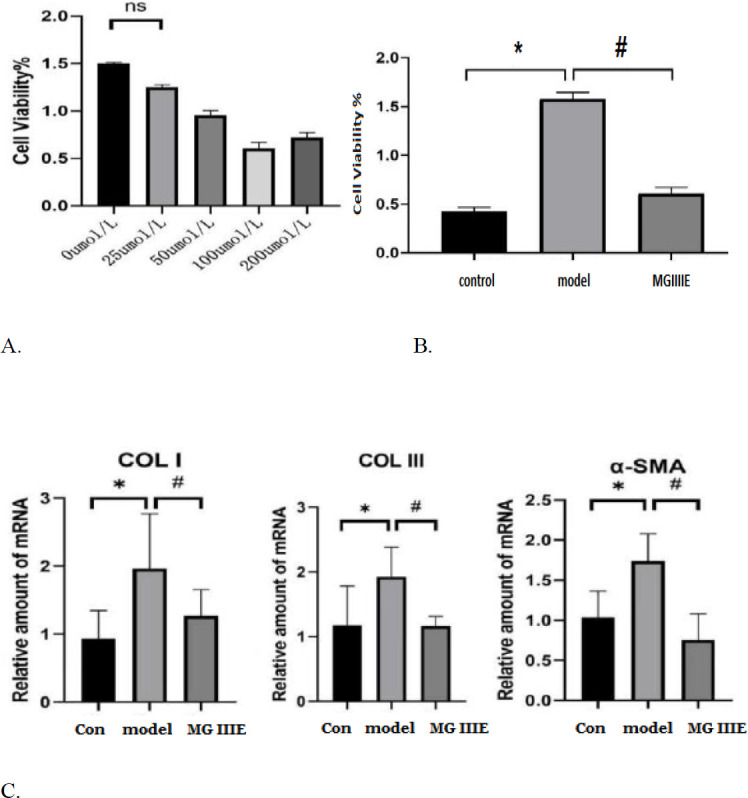
Results of *in vitro* experiments

A. The pre-experiment screened out the drug concentration of MGIIIE. 100 mol/l MGIIIE could inhibit the proliferation of myocardial fibroblasts to the maximum. B. Effect of MGIIIE on myocardial fibroblasts proliferation. Cell proliferration was evaluated using the CCK-8 assay. ^#^*P<*0.05 vs. the model group (n=3). C. Realtime qRT-PCR results. *P<0.05 shows significant differences between the indicated groups. ^#^*P<*0.05 show significant differences between the indicated groups (n=3)

**Figure 5:**
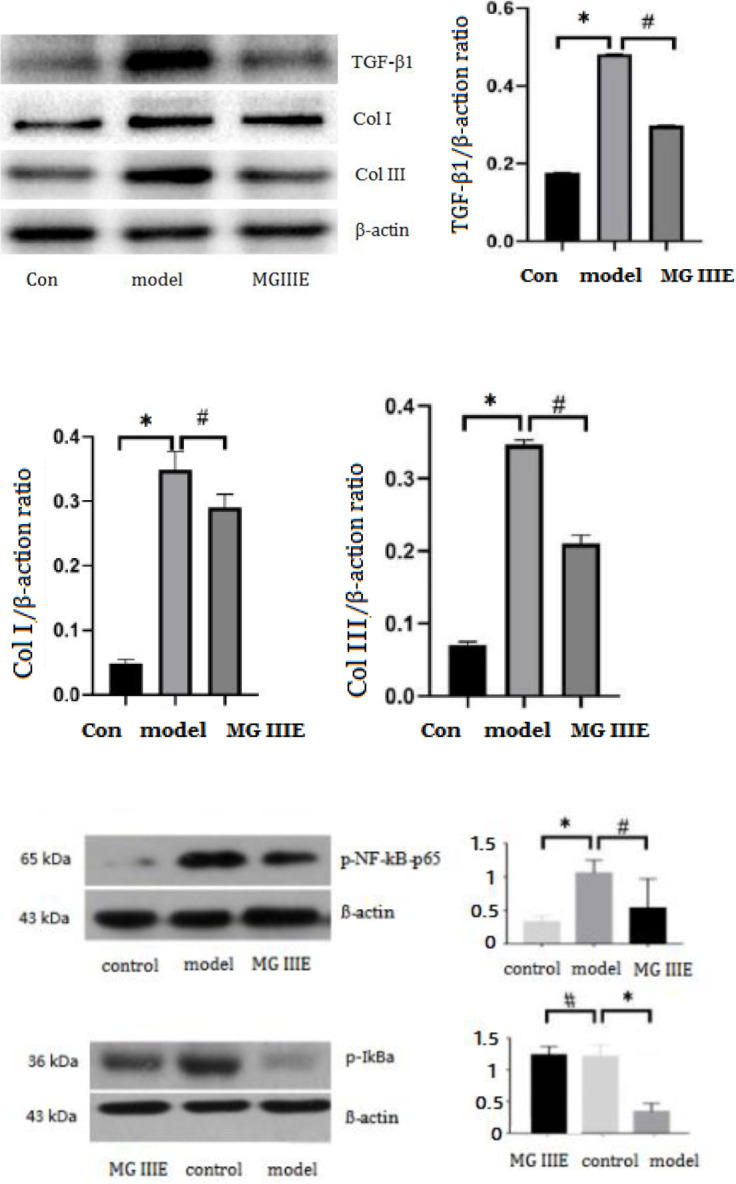
Effects of MGIIIE on protein expression in fibroblasts. *P<0.05 vs. the model group; ^#^*P<*0.05 vs. the MG IIIE group

**Figure 6:**
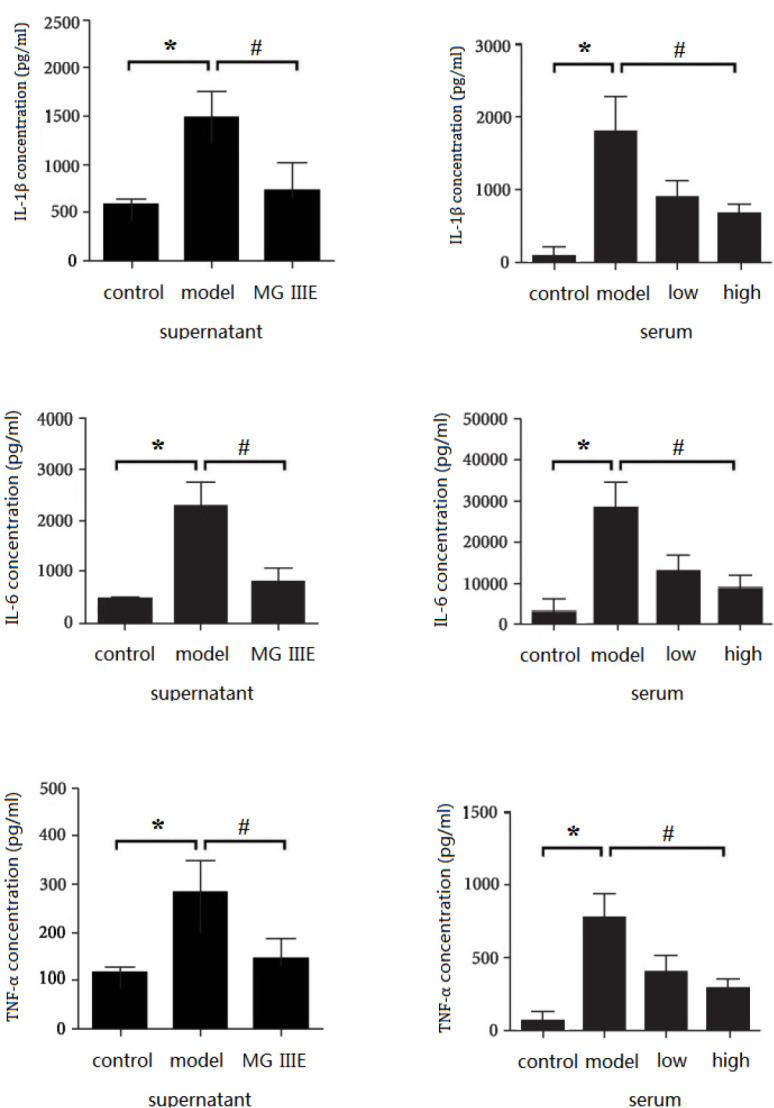
ELISA results. MGIIIE inhibits ISO-induced inflammatory response in the cardiac fibroblasts supernatant and the serum of mice. The level of IL-1, IL-6 and TNF-α was checked by ELISA. * *P<*0.05 versus the model group; ^#^ *P<*0.05 versus the model group

## Discussion

As part of the innate immune response, TLR4 can induce the activation and release of inflammatory and fibrotic factors through specific signal transduction pathways (26-30). Previous studies have reported that TLR4 is involved in promoting myocardial inflammation and ischemia/reperfusion injury, and aggravating cardiac remodeling and heart failure (31-34). TLR4 knockdown has been shown to reduce myocardial inflammation and fibrosis (35-37). In the present study, histopathological examination revealed significant inflammatory cell infiltration and fibrosis in the models of ISO-induced myocardial injury. The Western blot results showed that the expression of TLR4, MyD88, NF-κB, TGF-β1, and α-SMA in the model group was increased. This finding suggested that the TLR4/MyD88/NF-κB signaling pathway was involved in ISO-induced myocardial inflammation and fibrosis.

After 2 weeks of intragastric administration, MGIIIE was found to reduce the level of inflammation and fibrosis in the myocardium. To further investigate the potential mechanism by which MGIIIE alleviates myocardial fibrosis, we explored the expression of the TLR4-MyD88-NF-κB signaling pathway. Following ligand recognition, TLR4 recruits downstream signaling molecules containing the Toll-interleukin (IL)-1 receptor (TIR) domain, including MyD88, TIRAP containing TIR domain connexin (TIRAP), inducible interferon receptor (TRIF), and TRIF associated connexin (TRAM) (38-43). MyD88 is a signaling molecule required by most members of the TLR and IL-1 receptor family (44). In response to stimulation with LPS and other DAMPs, the TIR domain of TLR4 binds to MyD88 and activates IL-1 receptor-associated kinase (IRAK), TGF-β1-activated kinase 1 (TAK1), NF-κB inhibitor protein kinase (IKK), and eventually NF-κB (45-48). 

In this study, results showed that the level of TLR4, MyD88, and p-NF-κB protein expression was decreased in the MGIIIE group. These results suggested that the TLR4/MyD88/NF-κB signaling pathway may be one of the targets of MGIIIE to improve myocardial injury and fibrosis. In addition, we found that MGIIIE increased the expression of phosphorylated IκB, which may represent a mechanism by which MGIIIE reduced NF-κB transcriptional activity. At baseline, the NF-κB subunit dimer was bound by an NF-κB inhibitor (IκB). A protein kinase, termed IκB kinase (IKK), phosphorylates IκB, causing it to undergo proteasome degradation and release NF-κB subunit dimers into the nucleus, where they function as transcription factors for various inflammation-related genes (Figure 8) (49-53). Our results showed that there were low levels of IκB phosphorylation in AngII-treated fibroblasts and MGIIIE treatment increased the level of p-IκB. MGIIIE treatment decreased the expression of NK-κB stimulated by Ang II.

Increased TGF-β1 and α-SMA expression is a characteristic of cardiac fibrosis, which was also decreased in the MGIIIE group. Fibroblasts do not express α-SMA in healthy neonatal hearts (54). During the process of myocardial injury and fibrosis, myocardial fibroblasts are transformed into activated fibroblasts under the influence of pro-fibrosis factors, namely myofibroblasts. Myofibroblasts express α-SMA** (**55,56), a marker of fibroblast activation. In the present study, α-SMA protein expression was found to be increased in the ISO group, indicating that fibroblasts were activated into myofibroblasts. The latter produces a large amount of ECM (Col I and Col III), which exhibits excessive accumulation in the intercellular stroma and leads to myocardial fibrosis. Similar results have been observed in previous studies. For example, different doses of MGIIIE have been found to inhibit the expression and activity of inflammatory cytokines in LPS-induced lung injury models in a dose-dependent manner prior to LPS treatment in mice (57-59). 

## Conclusion

Collectively, these results indicate that MGIIIE has both anti-inflammatory and anti-fibrosis effects. It inhibited the activation of related signal molecules in the TLR4/MyD88/NF-κB pathway and decreased the levels of IL-1β, IL-6, and TNF-α. Moreover, MGIIIE can reduce the deposition of ECM and play a role in anti-myocardial fibrosis, which may provide novel options for the treatment of heart failure.

Of course, there are considerable deficiencies in this study. For example, only α-SMA was detected as a marker for the activation of cardiac fibroblasts into myofibroblasts. The cardiac myofibroblast was not identified directly. In future research, the effect of MGIIIE on the migration and contraction function of myofibroblast should also be tested. Another significant disadvantage is that we only detected the expression change of the TLR4-MyD88-NF-κB signal pathway, but did not use specific blockers/agonists to inhibit or promote this signal pathway, so as to further explore the role of MGIIIE on this signal pathway.

## Authors’ Contributions

YS performed the animal experiments, BL and ZM made analysis and interpretation and wrote this manuscript before submission. SS performed the fibroblasts experiment. WT performed the western blot to analyze the expression of related proteins. WL designed this study and provided financial support.

## Conflicts of Interest

The writers declare no conflicts of interest associated with the present manuscript.
